# Role of metabolic transformation in cancer immunotherapy resistance: molecular mechanisms and therapeutic implications

**DOI:** 10.1007/s12672-025-02238-3

**Published:** 2025-04-02

**Authors:** Sandesh Shende, Jaishriram Rathored, Tanushree Budhbaware

**Affiliations:** Central Research Laboratory and Molecular Diagnostics, School of Allied Health Sciences, Datta Meghe Institute of Higher Education and Research, Sawangi (Meghe), Wardha, 442001 Maharashtra India

**Keywords:** Cancer metabolism, Immunotherapy resistance, Tumour microenvironment, Glycolysis, Amino acid metabolism, Immune checkpoint inhibitors

## Abstract

**Background:**

Immunotherapy in the treatment of cancer, with immune inhibitors helps in many cancer types. Many patients still encounter resistance to these treatments, though. This resistance is mediated by metabolic changes in the tumour microenvironment and cancer cells. The development of novel treatments to overcome resistance and boost immunotherapy's effectiveness depends on these metabolic changes.

**Objective:**

This review concentrates on the molecular mechanisms through which metabolic transformation contributes to cancer immunotherapy resistance. Additionally, research therapeutic approaches that target metabolic pathways to enhance immunotherapy for resistance.

**Methods:**

We used databases available on PubMed, Scopus, and Web of Science to perform a thorough review of peer-reviewed literature. focusing on the tumor microenvironment, immunotherapy resistance mechanisms, and cancer metabolism. The study of metabolic pathways covers oxidative phosphorylation, glycolysis, lipid metabolism, and amino acid metabolism.

**Results:**

An immunosuppressive tumour microenvironment is produced by metabolic changes in cancer cells, such as dysregulated lipid metabolism, enhanced glutaminolysis, and increased glycolysis (Warburg effect). Myeloid-derived suppressor cells and regulatory T cells are promoted, immune responses are suppressed, and T cell activity is impaired when lactate and other metabolites build up. changes in the metabolism of amino acids in the pathways for arginine and tryptophan, which are nutrients crucial for immune function. By enhancing their function in the tumour microenvironment, these metabolic alterations aid in resistance to immune checkpoint inhibitors.

**Conclusion:**

Metabolic change plays a key role in cancer immunotherapy resistance. Gaining knowledge of metabolic processes can help develop efficient treatments that improve immunotherapy's effectiveness. In order to determine the best targets for therapeutic intervention, future studies should concentrate on patient-specific metabolic profiling.

## Introduction

Cancer major global health challenge, one of the complex diseases known to medicine [1]. Advances in cancer treatment, it continues to account for deaths worldwide. Various treatment strategies like immunotherapy have revolutionized cancer care [2]. Therapies such as immune checkpoint inhibitors (ICIs), chimeric antigen receptor (CAR) T-cell therapies, and cancer vaccines have shown results hope survival in patients with advanced malignancies [3]. These therapies work by stimulating the immune system to recognize and destroy tumour cells, providing treatment for cancer patients. However, these advancements, many patients fail to respond to immunotherapy, or their initial response decreases over time. This phenomenon, known as immunotherapy resistance, important in oncology and barrier to therapeutic potential of immunotherapy. Understanding the mechanisms of resistance is essential for the development of more effective treatments [4].

Cancer cells undergo metabolic changes in response to immunotherapy. Changes in the metabolic processes of cancer cells allow them to grow, produce energy, and endure in environments deficient in nutrients. These metabolic modifications include increased glutaminolysis, changes in lipid metabolism, and glycolysis (also known as the Warburg effect) [5]. The tumour microenvironment (TME) is a complex includes stromal cells, immune cells, extracellular matrix, and soluble factors. Immunotherapy’s therapeutic attempts and the metabolic alterations in cancer cells and the TME that facilitate immune suppression [6]. The reprogrammed metabolism of cancer cells contributes to an immunosuppressive microenvironment that impairs the immune system’s ability to effectively target and eliminate tumour cells [7]. Natural killer (NK) cells, cytotoxic T-cells, and other effector immune cells that regulate tumour growth are among the immune cells whose function is suppressed by the buildup of metabolites such as lactate and other byproducts of glycolysis. TME that suppresses the immune system makes immunotherapy successful [8].

Cancer cells have long undergone metabolic changes. The Warburg effect was first identified by Otto Warburg in the 1920s, when he reported that cancer cells preferred aerobic glycolysis to oxidative phosphorylation when oxygen was present. This finding contributes to our understanding of how cancer alters metabolic pathways [9]. Research revealed that cancer cells manipulate metabolic pathways, such as fatty acid oxidation, amino acid metabolism, and mitochondrial dynamics, to support their growth and survival [10]. According to recent research, these metabolic alterations promote the growth of tumours and the emergence of immunotherapy resistance. Research on the relationship between immune mechanisms and cancer metabolism provides insight into the pathophysiology of cancer and its treatment resistance.

Immunotherapy resistance lies between cancer cells and immune cells within the altered TME [11]. The accumulation of lactate, a byproduct of the Warburg effect important for tumour progression. Lactate support tumour growth by providing energy and activity of effector immune cells such as T-cells and NK cells [12]. Lactate making them less effective at targeting and eliminating tumour cells. Depletion of essential amino acids, such as tryptophan and arginine, by tumour-associated enzymes like indoleamine 2,3-dioxygenase (IDO) and arginase, contributes to immune suppression by starving immune cells [13]. These metabolic alterations reduce nutrients that required for the proper functioning of T-cells, inhibiting effective immune responses. Dysregulation in lipid metabolism like fatty acid oxidation in regulatory T cells, important for promoting an immunosuppressive environment. Tregs, which suppress immune responses altered metabolic state. These metabolic changes provide an adverse environment that weakens the immune system and reduces the effectiveness of immunotherapies [14].

The possibility of targeting metabolic pathways to reverse immunosuppressive effects and enhance the effectiveness of immunotherapies. Inhibitors of lactate dehydrogenase (LDH), an enzyme involved in lactate production, and IDO inhibitors [15]. These treatments enhance the effectiveness of ICIs by restoring immune function and lessening the immunosuppressive effects of lactate. There are obstacles in integrating these treatments into standard clinical practice. The metabolic heterogeneity seen in various tumours is one of the main obstacles. Understanding the metabolic changes in tumours is necessary to target the metabolic features that each tumour displays. Successful clinical application is also hampered by the toxicity of metabolic inhibitors and the fact that they target metabolic pathways without harming healthy, normal cells. Therapeutic results are complicated by the interactions between immune cells and cancer cells within the TME because immune cell function may be impacted by treatments that target tumour metabolism [16].

This review offers a thorough examination of the relationship between immunotherapy resistance and metabolic transformation in cancer. Examine the molecular mechanisms through which immunotherapies are suppressed and immune protection is mediated by altered metabolism. Furthermore, therapeutic approaches that focus on metabolic pathways can help overcome resistance and enhance immunotherapy results. Through knowledge synthesis and research gap identification, this review offers insight into using immunotherapy to target cancer metabolism. Moreover, metabolic heterogeneity of tumours and their microenvironments is provided by sophisticated technologies like transcriptomics, single-cell metabolomics, and others. By identifying metabolic targets and assisting with therapeutic strategies, these technologies offer more individualised and efficient cancer treatments.

In conclusion, developing new therapeutic strategies requires an understanding of the role metabolic transformation plays in cancer and how it affects resistance to immunotherapy. TME, affect immunological responses, boost immunotherapy effectiveness, and enhance patient outcomes. To help cancer patients around the world, it is crucial to close the gap between basic research and clinical application. Contribute to initiatives to enhance cancer care and open up new research avenues with this review.

### Molecular mechanisms of metabolic transformation in cancer

Metabolic transformation of cancer cells that survive, proliferate, and immune responses in environments. Reconfiguration of glucose, amino acid, and lipid metabolism to support tumour progression and immunosuppression.

**Fig. 1:**
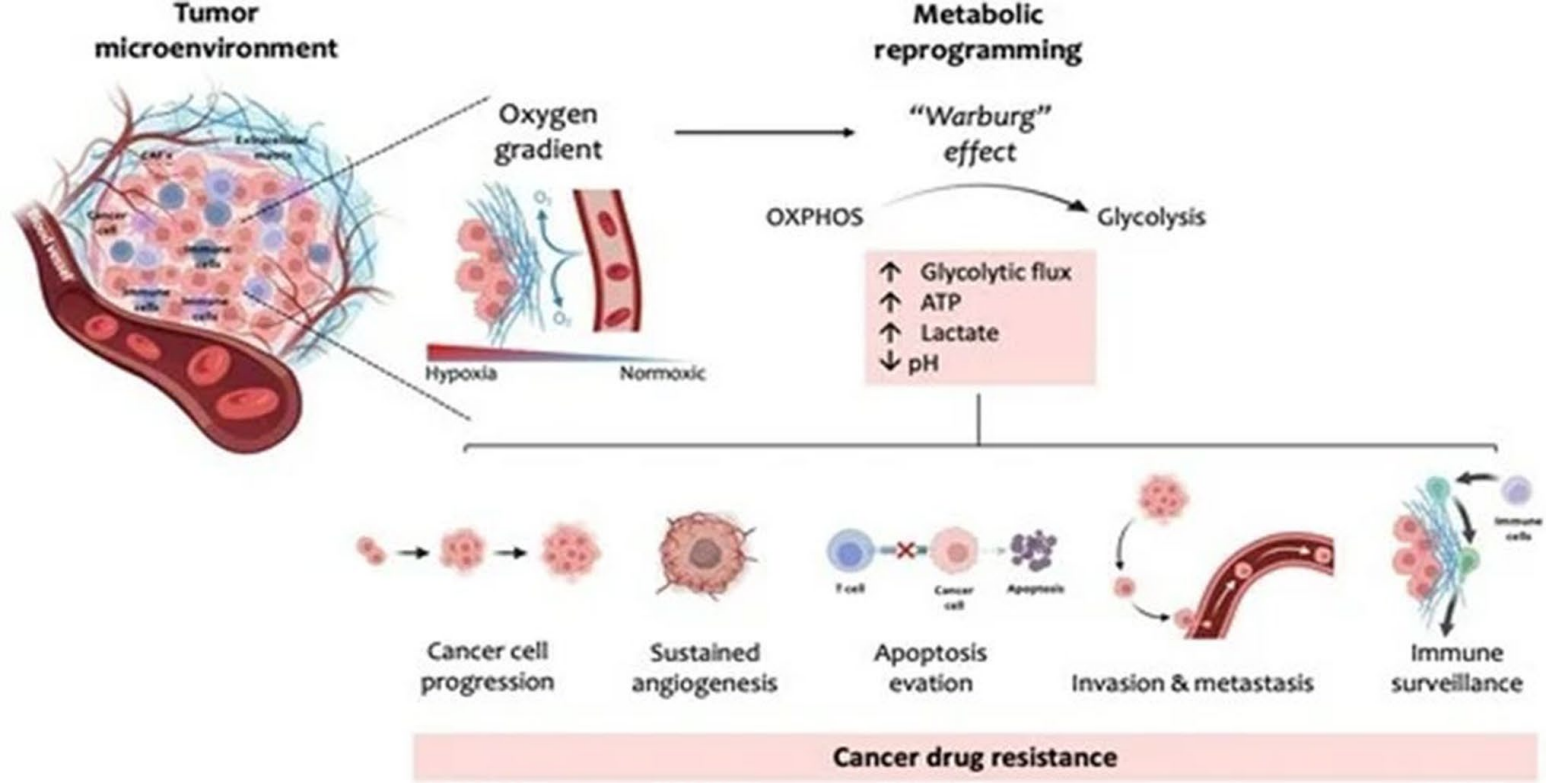
Warburg effect of cancer (Adapted from Cunha et al.) [20]

The Warburg effect of cancer, involves cancer cells aerobic glycolysis over oxidative phosphorylation. This alteration of cancer cells generate energy and produce building blocks for cell growth [17]. Enzymes, such as Hexokinase 2 (HK2), Pyruvate Kinase M2 (PKM2), and LDH, are regulates in tumours to accelerate glycolysis and lactate production [18]. The accumulation of lactate in the TME leads to acidification, which impairs immune cell functions like T-cells and NK cells while promoting immune-suppressive cell recruitment. Targeting glycolytic enzymes, such as LDH inhibitors, is being explored as a therapeutic strategy to overcome immunosuppression and enhance the effectiveness of immunotherapies [19]. In Fig. 1 shows the how TME metabolic reprogramming through Warburg Effect in Cancer drug resistance.

Cancer cells undergo metabolic transformation of amino acids, including tryptophan, arginine, and glutamine, to evade immune responses [21]. In tryptophan metabolism, the enzyme IDO converts tryptophan into kynurenine, which suppresses T-cell function and promotes regulatory T-cell activity, allowing tumour survival. In arginine metabolism, arginase, overexpressed in tumour-associated macrophages and myeloid-derived suppressor cells (MDSCs), depletes arginine, which is vital for T-cell activation, weakening immune responses [22]. In glutamine metabolism, glutaminase (GLS) upregulation in tumours alters immune cell activity, further enhancing immune evasion. Inhibiting IDO, arginase, or GLS offers therapeutic potential to restore immune cell function and improve the effectiveness of ICIs [23].

Lipid metabolism is significantly altered in cancer cells, contributing to tumour progression and immune suppression. Fatty acid oxidation (FAO) is enhanced in Tregs and tumour-associated macrophages (TAMs), promoting their immunosuppressive functions [24]. Carnitine palmitoyl transferase 1A (CPT1A) is a key enzyme involved in FAO, and its inhibition has shown promise in disrupting Treg-mediated immune suppression [25]. By overexpressing lipid transporters such as cluster of differentiation (CD36), cancer cells also enhance lipid uptake, promoting membrane biosynthesis and energy storage while preventing immune cell activation. Further encouraging resistance to immunotherapy is the upregulation of cholesterol biosynthesis in tumours, which leads to T-cell exhaustion. By focussing on these lipid metabolic pathways, anti-tumor immunity and treatment results may be enhanced [26].

Hypoxia, commonly present in solid tumours, triggers the stabilization of hypoxia-inducible factor 1 alpha (HIF-1α), which regulates various metabolic and immune responses. HIF-1α induces the expression of glycolytic enzymes and immune checkpoint molecules like PD-L1, promoting immune evasion [27]. Hypoxia also causes lactate accumulation, exacerbating immunosuppression by limiting immune cell infiltration and function. The abnormal blood vessels formed under hypoxic conditions hinder immune cell access to the tumour. Therapeutic strategies targeting HIF-1α or normalizing tumour vasculature are being investigated to improve immune cell infiltration and the efficacy of immunotherapies [28].

Mitochondrial transformation in cancer cells influences both metabolism and immune response. While many cancers rely on glycolysis, some tumours depend on oxidative phosphorylation (OXPHOS) for energy production [29]. Reactive oxygen species (ROS) produced by high mitochondrial activity in cancer cells inhibit T-cell function and increase the expression of immune checkpoints. The survival of cancer cells and their resistance to immune-mediated apoptosis are also influenced by altered mitochondrial dynamics, including fission and fusion. Immunotherapy resistance may be overcome and immune responses against tumours strengthened by focussing on mitochondrial metabolism, for example, by employing ROS scavengers or OXPHOS inhibitors [30].

Epigenetic modifications, such as DNA methylation and histone acetylation, regulate the expression of metabolic genes in cancer, impacting immune evasion. Oncometabolites like 2-hydroxyglutarate (2-HG) influence gene expression patterns that promote tumour survival and immune tolerance [31]. Targeting these epigenetic regulators in combination with metabolic inhibitors presents a potential strategy to reverse immunosuppression and improve the efficacy of cancer immunotherapies. This approach could provide a synergistic effect, enhancing the overall response to treatment by overcoming immune resistance mechanisms [32].

### Impact of metabolic transformation on immunotherapy resistance

Cancer cells undergo metabolic transformation to support their growth and survival. This metabolic shift not only facilitates tumour progression but also suppresses immune responses, leading to challenges in immunotherapy [33]. The TME produced by these metabolic alterations restricts the efficacy of treatments like ICIs that depend on immune activation. This article looks at how metabolic transformation affects antigen presentation, immune cell functions, and immune suppression, all of which lead to resistance to immunotherapy [34].

In the TME, immune cells like cytotoxic T lymphocytes (CTLs) and NK cells, essential for attacking tumour cells, face exhaustion due to nutrient scarcity and toxic metabolic byproducts [35]. Cancer cells rely on aerobic glycolysis for energy, which depletes glucose, a critical nutrient for immune cells, impairing CTL function. Additionally, tryptophan metabolism by tumour cells creates kynurenine, further suppressing T-cell activity and promoting regulatory T-cell (Treg) functions, exacerbating immune exhaustion [36]. Moreover, amino acids like glutamine and arginine, important for T-cell activation, are also depleted, further compromising immune responses and immunotherapy effectiveness [37].

Metabolic transformation in cancer cells also promotes the upregulation of immune checkpoints, which hinder the immune system's ability to combat tumours [38]. HIF1α, activated under low oxygen conditions, stabilizes and upregulates immune checkpoint molecules like PD-L1. This reduces the activity of T-cells by binding to PD-1 receptors and suppressing immune responses [39]. Additionally, lactate, produced from glycolysis, accumulates in the TME, further amplifying immune checkpoint signalling and preventing effective T-cell activity, posing a significant barrier to immunotherapy [40].

Metabolic transformation not only impacts immune cells but also enhances the recruitment of immunosuppressive cells, such as Tregs, MDSCs, and TAMs, that contribute to an immunosuppressive environment [41]. Metabolic byproducts such as lactate and kynurenine are used by tumour cells to draw in Tregs, which inhibit antitumor immune responses. FAO promotes Treg function and survival even more. Similarly, TAMs lean towards an M2 phenotype, which reinforces immune suppression, while MDSCs, which are activated by lactate and hypoxia, suppress T-cell responses through processes like arginine depletion and ROS production [42].

Effective antigen presentation by dendritic cells (DCs) is crucial for initiating antitumor immunity [43]. However, the metabolic transformation in cancer cells disrupts this process. Lipid accumulation in DCs due to altered metabolism interferes with antigen processing, impairing T-cell activation [44]. Similarly, TAMs exhibit impaired antigen presentation, which diminishes the immune system’s ability to recognize and attack tumour cells, hindering the success of immunotherapies such as cancer vaccines and adoptive T-cell therapies [45].

**Fig. 2:**
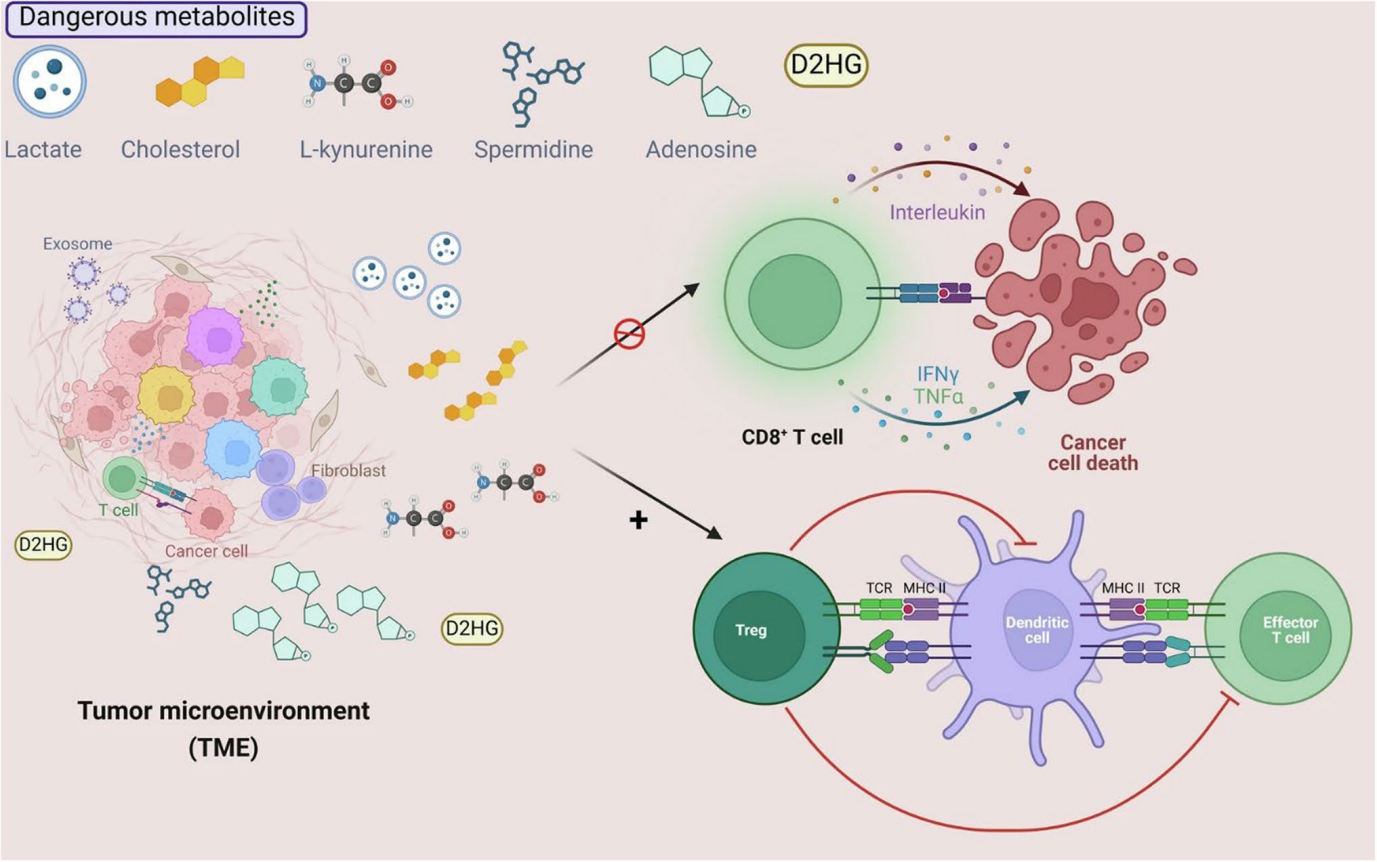
Tumor microenvironment and dangerous metabolites (Adapted from Zeng et. al.) [48]

When solid tumours experience hypoxia, HIF1α, which controls angiogenesis and glycolysis, stabilises, causing metabolic transformation. These modifications produce more lactate and less oxidative phosphorylation, which makes the environment unfriendly to immune cells [46]. Hypoxia-driven angiogenesis alters TME architecture, leading to irregular blood vessels that prevent immune cells from infiltrating tumours. This "immune exclusion" reduces the efficacy of immunotherapies like ICIs and adoptive T-cell therapies by limiting immune cell access to tumour cells [47]. The innate immune system's most important cells, macrophages, can develop into two subtypes depending on various stimuli in the microenvironment: M1 macrophages, which are triggered by IFN-γ/lipopolysaccharides (LPS), and M2 macrophages, which are triggered by IL-4/IL-10/IL-13. These subgroups differ completely in their functional traits and molecular manifestations. To differentiate themselves from macrophages, tumor-associated macrophages (TAMs) exhibit a number of traits in the tumor microenvironment (TME). Similar in appearance and function to M2 macrophages, TAMs can produce a range of cytokines to promote tumor growth. Understanding immune escape mechanisms requires a deep understanding of pro-tumor and antitumor metabolic alterations in TAM, since both tumor cells and macrophages can use metabolic reprogramming to meet the energy requirements for rapid cell growth and proliferation. Figure 2 shows the how dangerous metabolites interacting with TME.

To overcome resistance, targeting the metabolic transformation pathways in cancer cells may enhance immunotherapy effectiveness. Inhibiting key glycolytic enzymes like HK2 and LDH can reduce lactate accumulation, restoring immune cell function and enhancing antitumor responses [49]. Furthermore, tryptophan and arginine depletion can be avoided by inhibiting enzymes involved in amino acid metabolism, such as IDO and arginase, which supports T-cell function. While addressing hypoxia with HIF1α inhibitors or anti-angiogenesis therapies may improve immune cell infiltration, overcoming the immune exclusion effect and enhancing the effectiveness of immunotherapies, targeting lipid metabolism with FAO inhibitors can decrease the immunosuppressive activity of Tregs and TAMs [50].

### Therapeutic implications and emerging strategies

Metabolic transformation in cancer cells plays a pivotal role in tumour progression and immune evasion. Tumour cells manipulate the metabolic environment within the TME, creating conditions that suppress immune responses and reduce the effectiveness of immunotherapies [50]. These metabolic changes, however, also provide opportunities for therapeutic intervention. Emerging strategies aimed at targeting these altered metabolic pathways offer potential to overcome resistance to immunotherapy and enhance therapeutic outcomes by restoring immune function and improving the efficacy of cancer treatments [51].

**Fig. 3:**
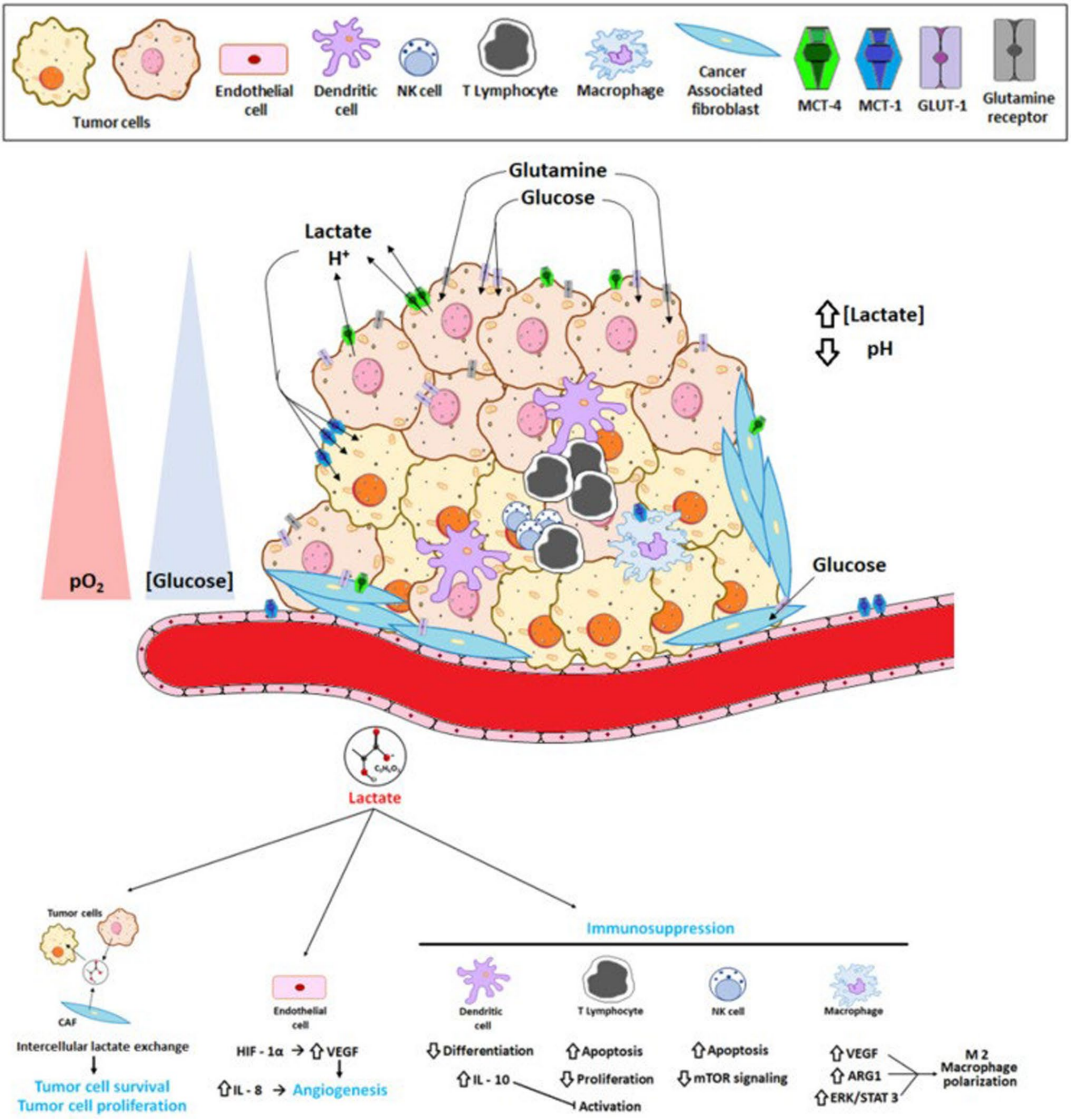
Effect of lactate on the TME (Adapted from Pérez-Tomás R et. al.) [12]

Cancer cells heavily rely on glycolysis, even in oxygen-rich conditions (known as the Warburg effect), to support tumour growth and immune evasion [52]. Glycolysis generates a lactate-rich, acidic environment in the TME, which hampers the function of immune cells. Targeting this metabolic pathway holds promise in restoring immune function and enhancing therapy. LDH inhibitors, such as FX11, have demonstrated the potential to reduce lactate production, thus improving T-cell activity [53]. Inhibiting HK2, which catalyses the first step of glycolysis, can disrupt glucose metabolism in cancer cells and promote better immune responses by reducing nutrient competition between immune cells and tumour cells. Figure 1 Shows the lactate effect on TME. These glycolysis inhibitors could improve the efficacy of immunotherapies by restoring glucose availability and counteracting the immunosuppressive effects of lactate. [54] (Fig. 3).

Amino acid metabolism is essential for the survival of both cancer and immune cells. Tumour cells deplete critical amino acids like tryptophan, arginine, and glutamine in the TME through enzymes such as IDO, arginase, and glutaminase, which suppress immune activity. Targeting these metabolic pathways can reverse immune suppression [55]. IDO inhibitors, such as epacadostat, aim to restore tryptophan levels and reduce kynurenine-driven immune suppression. Similarly, arginase inhibitors like INCB001158 can restore T-cell activity by increasing arginine levels, and glutaminase inhibitors such as CB839 may help provide effector T-cells with the necessary glutamine for enhanced immune function. These strategies are promising for overcoming immune exhaustion and enhancing antitumor immunity, especially when combined with ICIs [56].

Lipid metabolism is a crucial factor in immune suppression within the TME. Tumour cells, along with immune-suppressive cells such as regulatory T-cells and TAMs, rely on lipid metabolism for their survival and function [57]. FAO inhibitors, like etomoxir, target this metabolic pathway, transformation TAMs to a pro-inflammatory phenotype and reducing the suppressive activity of Tregs. Additionally, cholesterol accumulation impairs DC function, reducing their ability to activate T-cells. Cholesterol-modulating therapies, such as statins, may enhance DC function and improve overall immune responses. Targeting lipid metabolism can offer dual benefits by directly disrupting tumour metabolism while also reinvigorating immune cell function, particularly in tumours enriched with lipid-storing suppressor cells [58].

Hypoxia, common in solid tumours, triggers metabolic transformation and immune suppression through the stabilization of HIF1α. Inhibiting HIF1α, using compounds like acriflavine, can normalize the TME by reducing hypoxia-induced immune suppression and enhancing T-cell infiltration and activity [59]. Additionally, hypoxia-activated prodrugs (HAPs) are designed to be activated in low oxygen conditions, selectively targeting hypoxic tumour regions while sparing normal tissues. These therapies can work synergistically with immune checkpoint inhibitors to reduce hypoxia-driven immune suppression. Targeting hypoxia within the tumour is an effective strategy to remodel the TME, improving immune responses and the overall efficacy of cancer treatments [60].

Combination therapies are emerging as an effective approach to enhance immunotherapy outcomes by addressing the complexity of metabolic transformation and immune suppression. Combining IDO inhibitors with anti-PD1 antibodies can provide a synergistic effect by reducing immunosuppressive metabolites and boosting T-cell activity [61]. Similarly, FAO inhibitors can lessen Treg-mediated suppression when used in conjunction with adoptive cell therapies like CAR T-cell therapy, increasing the effectiveness and durability of CAR T-cells. Furthermore, by lessening lactate-mediated dendritic cell suppression, glycolysis inhibitors in conjunction with cancer vaccines can improve antigen presentation and T-cell activation. Targeting several levels of immune suppression in order to overcome resistance and enhance treatment results makes these combination strategies a promising avenue for the future [62].

### Challenges and future perspectives

Tumours exhibit metabolic diversity, influenced by factors such as tissue type, genetic mutations, and the TME. Different subpopulations within the same tumour may rely on various metabolic pathways like glycolysis, oxidative phosphorylation, or fatty acid oxidation for survival. This metabolic plasticity enables tumours to adapt to conditions like hypoxia and nutrient scarcity, contributing to therapeutic resistance [63]. To tackle this challenge, personalized medicine approaches are crucial, using advanced diagnostic tools like metabolomic profiling and genomic sequencing to identify tumour-specific metabolic dependencies. Tailored therapies can then target these vulnerabilities, improving treatment outcomes while minimizing side effects [64].

Selective targeting of tumour metabolism remains a challenge due to shared metabolic pathways between cancer and normal cells. Inhibiting pathways like glycolysis or glutamine metabolism may inadvertently affect normal, rapidly dividing cells, leading to systemic toxicity [65]. To address this, researchers are developing selective inhibitors targeting cancer-specific enzymes, such as LDHA, which is overexpressed in tumours but absent in normal tissues. Additionally, the use of prodrugs activated only in the TME, along with optimized dosing regimens and combination therapies, can reduce off-target effects while enhancing the therapeutic impact of metabolic inhibitors [66].

Cancer cells have the ability to adapt quickly to metabolic therapies, bypassing blocked metabolic pathways by activating alternative routes. For example, inhibiting glycolysis may prompt tumours to rely more on oxidative phosphorylation or fatty acid oxidation. This flexibility challenges the long-term effectiveness of metabolic inhibitors [67]. Combination therapies are being explored as a solution, such as pairing metabolic inhibitors with ICIs or dual inhibition of complementary pathways like glycolysis and oxidative phosphorylation. These strategies aim to prevent resistance and enhance the overall efficacy of treatment [68].

Despite promising preclinical results, translating metabolic therapies into clinical practice presents significant challenges, such as developing robust clinical trial designs and identifying predictive biomarkers [69]. Clinical trials need to account for the metabolic heterogeneity of tumours, and the lack of reliable biomarkers complicates patient selection. Biomarker-driven patient stratification is essential to ensure that treatments are tailored to those most likely to benefit. Additionally, novel endpoints beyond traditional tumour shrinkage measures are necessary to evaluate therapeutic efficacy, such as assessing changes in tumour metabolism or immune activation within the TME [70].

Emerging technologies provide exciting prospects to address the challenges of targeting metabolic transformation in cancer. Single-cell metabolomics allows for the analysis of individual cell metabolism, providing detailed insights into tumour heterogeneity [71]. Spatial transcriptomics maps gene expression within the tumour and its microenvironment, revealing how metabolic interactions influence therapy resistance. Furthermore, AI and machine learning can analyse complex datasets, identifying novel metabolic targets and predicting therapeutic responses, thus accelerating drug discovery and optimizing clinical trial designs [72].

The future of metabolic-targeted cancer therapy lies in integrating these strategies with existing treatments. Key areas of focus include the development of reliable biomarkers to predict metabolic dependencies and monitor treatment responses, ensuring that therapies are administered to the right patients [73]. Technological developments in drug delivery and nanotechnology will allow metabolic inhibitors to be more precisely targeted to tumours while causing the least amount of harm to healthy tissues. Overcoming resistance may be greatly aided by combining metabolic therapies with immunotherapies such as ICIs or CAR T-cell therapies. Lastly, in order to optimise treatment plans and enhance patient outcomes, it will be essential to comprehend the wider systemic effects of metabolic therapies, including their influence on the gut microbiota and immune system [74].

## Discussion

The Warburg effect, a key feature of cancer cells, is characterized by increased aerobic glycolysis even in the presence of oxygen [52]. Rapid ATP synthesis and the biosynthesis of molecules necessary for tumour growth are made possible by this metabolic shift. But the elevated glycolysis causes lactate to build up, which lowers the TME's pH. This acidic environment encourages the recruitment of immunosuppressive cells such as regulatory T cells and MDSCs while suppressing the activity of CTLs and NK cells. Thus, the Warburg effect complicates the efficacy of immunotherapies by promoting tumour growth and immune evasion [9]. Table 1 shows recent studies with advantages and disadvantages related to metabolic transformation in cancer immunotherapy resistance.

**Table 1:** Recent Studies with Merits and Demerits related to metabolic transformation in cancer immunotherapy resistance

Author Name and Year	Findings of the Study	Merits	Demerits
Smith et al. 2016 [75]	Demonstrated that the Warburg effect suppresses Tcell function by increasing lactate production in the TME	Highlighted lactate’s role in immune evasion	Focused primarily on in vitro models, lacking clinical validation
Johnson et al.2016 [76]	Identified IDO1mediated tryptophan metabolism as a key mechanism of Tcell suppression	Proposed IDO1 inhibitors as a therapeutic target	Limited to preclinical data without in vivo efficacy studies
Zhang et al.2017 [77]	Revealed how lipid accumulation in TAMs promotes an immunosuppressive phenotype	Established a link between lipid metabolism and immune suppression	Did not investigate combinatorial strategies with ICIs
Lee et al. 2017 [78]	Showed that hypoxia induced HIF1α upregulates PDL1 expression in tumours, leading to immunotherapy resistance	Provided evidence for hypoxia targeted therapies	Hypoxia targeted treatments may have off target effects
Wang et al.2018 [79]	Reviewed metabolic pathways that impair antigen presentation, such as lactate mediated dendritic cell dysfunction	Highlighted the interplay between metabolism and immune activation	Lacked experimental validation of proposed pathways
Martinez et al. 2019 [80]	Found that arginine metabolism via arginase1 suppresses Tcell activity in the TME	Proposed arginase inhibitors as an adjunct to ICIs	Did not explore combination strategies in detail
Huang et al. [81]	Identified metabolic competition in the TME as a key driver of Tcell exhaustion	Offered insights into nutrient deprivation induced immune suppression	Focused on conceptual frameworks without experimental data
Brown et al. 2021 [82]	Reported that kynurenine AhR signalling enhances Treg recruitment in the TME	Established kynurenine’s role in immune tolerance	Lack of studies on selective AhR inhibitors
Wilson et al.2021 [83]	Demonstrated that targeting HIF1α in combination with ICIs enhances antitumor immunity	Provided preclinical evidence for combination strategies	Did not evaluate long-term effects of HIF1α inhibition
Ahmed et al.2023 [84]	Reviewed clinical trials targeting metabolic pathways in combination with ICIs	Summarized ongoing translational efforts in metabolic therapies	Highlighted the limited success of metabolic inhibitors in advanced clinical stages

For cancer to undergo metabolic transformation, changes in amino acid metabolism are essential. Enzymes like indoleamine 2,3-dioxygenase (IDO1), which converts tryptophan to kynurenine, are upregulated in tumours. Tryptophan deficiency inhibits T cell activity and proliferation, whereas kynurenine increases immune tolerance by activating Tregs' aryl hydrocarbon receptor (AhR) [56]. Furthermore, tumours suppress antitumor immune responses by increasing glutamine metabolism to support their anabolic processes. These alterations in the metabolism of amino acids create an environment that restricts the ability of the immune system to effectively combat tumours, which leads to resistance to immunotherapy [23].

Lipid metabolism plays a critical role in immunotherapy resistance by affecting immune cell function. Tumours increase fatty acid synthesis, depleting essential fatty acids in the TME, which leads to the functional exhaustion of CTLs [24]. Furthermore, the immunosuppressive activity of MDSCs is enhanced by lipid accumulation. Additionally, TAMs adopt an M2-like phenotype, which promotes immunological evasion and tumour growth. These changes in lipid metabolism lower the effectiveness of immunotherapies and encourage the growth of tumours by creating an environment that is inhospitable to immune cells [57].

A common characteristic of the TME is hypoxia, which stabilises HIF1α and promotes metabolic transformation. HIF1α boosts the expression of immune checkpoint molecules like PDL1 and upregulates glycolytic enzymes, increasing lactate production. Furthermore, hypoxia-induced HIF1α activation hinders DC maturation and antigen-presenting ability and attracts immunosuppressive cells. The TME is a difficult environment for immunotherapy to work in because of the metabolic alterations brought on by hypoxia and immune evasion mechanisms [27].

Metabolic competition within the TME leads to the depletion of key nutrients such as glucose, amino acids, and oxygen, causing metabolic exhaustion in CTLs. This state of exhaustion impairs their ability to proliferate, produce cytokines, and carry out cytotoxic functions effectively [85]. Consequently, the effectiveness of ICIs is reduced in metabolically stressed TME. The lack of essential resources in the TME further exacerbates immune cell dysfunction, limiting the success of immunotherapies and allowing tumour progression [86].

Metabolic transformation directly influences the upregulation of immune checkpoint molecules that inhibit immune responses [50]. For example, elevated glycolysis and lactate accumulation in cancer cells are associated with increased PDL1 expression, which suppresses T cell activation. Similarly, alterations in lipid metabolism can upregulate inhibitory receptors such as TIM3 and LAG3 on immune cells, further dampening immune responses. These metabolic changes contribute to the development of immunotherapy resistance, as immune checkpoints prevent effective immune cell activation and antitumor responses [87].

The altered metabolic environment in the TME promotes the recruitment of immunosuppressive cells, which further complicates immunotherapy effectiveness. High levels of lactate, kynurenine, and other metabolites attract Tregs, MDSCs, and TAMs [88]. These cells produce inhibitory cytokines such as IL-10 and TGFβ, which suppress antitumor immunity. Additionally, MDSCs secrete molecules like arginase1 and nitric oxide, which inhibit T cell function, contributing to immune evasion and resistance to immunotherapy [89].

Initiating strong antitumor immune responses requires effective antigen presentation. On the other hand, antigen processing and presentation are disturbed by metabolic stress in the TME. Lactate buildup and hypoxia hinder DC maturation and function, which restricts their capacity to activate CTLs. As a result, immunotherapy has poor results and immune responses against the tumour are diminished. Therefore, the TME's changed metabolic state contributes to resistance by interfering with the vital process of antigen presentation in addition to impairing immune cell function [90].

Targeting glycolysis presents a promising strategy to overcome immunotherapy resistance. Inhibitors of glycolytic enzymes like hexokinase and LDH can reduce lactate production, restoring immune cell function [54]. Preclinical studies have shown that combining glycolytic inhibitors with ICIs enhances the antitumor immune response by improving T cell activity. This approach aims to reprogram the TME to a more immune-friendly environment, potentially improving the success of immunotherapy in resistant tumours [91].

Therapies that target amino acid metabolism, such as inhibitors of IDO1, can reverse immune suppression in the TME. IDO1 inhibitors block the catabolism of tryptophan, restoring T cell function and promoting antitumor immunity. Similarly, glutaminase inhibitors can disrupt glutamine metabolism, creating a metabolic environment that supports immune cell activity [32]. These strategies aim to improve immune responses by targeting the metabolic pathways that tumours exploit to evade immune surveillance.

Modulating lipid metabolism offers another potential approach to overcome immunotherapy resistance. Inhibitors of fatty acid synthase (FASN) and other lipid metabolic pathways can reduce lipid accumulation in MDSCs and TAMs, enhancing CTL function and reducing immunosuppressive effects [92]. By transformation the lipid metabolism in the TME, these therapies can support a more favourable immune environment and potentially improve the effectiveness of ICIs in cancer treatment [24].

Hypoxia-targeted therapies, such as hypoxia-activated prodrugs (HAPs) and HIF1α inhibitors, can help reverse immune suppression in the TME. These agents work by normalizing oxygen levels and reducing the effects of hypoxia-induced metabolic changes that promote immune evasion. Combining these therapies with ICIs may enhance immune responses and improve the efficacy of immunotherapy, offering a novel strategy to combat resistance driven by hypoxia in tumours [27].

Combination therapies that target multiple metabolic pathways alongside immunotherapy hold great promise in overcoming immunotherapy resistance. For instance, combining IDO1 inhibitors with glycolytic inhibitors or hypoxia-targeted therapies could address various aspects of resistance within the TME [39]. While these strategies offer potential, they require careful optimization to minimize systemic toxicity and metabolic heterogeneity. The goal is to create a more supportive immune environment by targeting the metabolic transformation of tumours, thereby enhancing the success of immunotherapy [11].

While targeting metabolic transformation presents a promising therapeutic avenue, several challenges remain. The metabolic heterogeneity of tumours, even within the same tumour, complicates the identification of universal targets. Furthermore, metabolic therapies must be carefully balanced to avoid adverse effects on normal tissues. Future research should focus on developing precision medicine approaches tailored to the specific metabolic profiles of individual tumours [93]. Finding biomarkers for patient selection and improving treatment plans will require developments in metabolomics, single-cell RNA sequencing, and imaging technologies. Novel combination strategies to overcome immunotherapy resistance will be made possible by an understanding of the dynamic interactions between immune cells and tumour metabolism [94].

## Conclusion

Metabolic transformation is a pivotal factor in cancer immunotherapy resistance, shaping an immunosuppressive tumour microenvironment that undermines the efficacy of immune checkpoint inhibitors. By unravelling the molecular mechanisms linking altered metabolic pathways to immune evasion, this review underscores the potential of targeting metabolic vulnerabilities to overcome therapeutic resistance. Future strategies should prioritize patient specific metabolic profiling and the integration of metabolic inhibitors with immunotherapies to achieve more durable and personalized treatment outcomes. As research progresses, this synergistic approach holds promise for transforming cancer care and improving the lives of patients worldwide.

## Data Availability

No datasets were generated or analysed during the current study.

## Abbreviations

ICIs: Immune Checkpoint Inhibitors
CAR: Chimeric Antigen Receptor
TME: Tumour Microenvironment
NK: Natural Killer
IDO: Indoleamine 2,3-dioxygenase
LDH: Lactate Dehydrogenase
HK2: Hexokinase 2
PKM2: Pyruvate Kinase M2
MDSCs: Myeloid-derived Suppressor Cells
GLS: Glutaminase
FAO: Fatty Acid Oxidation
TAMs: Tumour-associated Macrophages
CPT1A: Carnitine Palmitoyl Transferase 1A
CD36: Cluster of Differentiation
HIF-1α: Hypoxia-inducible Factor 1 alpha
OXPHOS: Oxidative Phosphorylation
ROS: Reactive Oxygen Species
2-HG: 2-Hydroxyglutarate
CTLs: Cytotoxic T lymphocytes
DCs: Dendritic cells
IDO1: Indoleamine 2,3-dioxygenase
AhR: Aryl Hydrocarbon Receptor
FASN: Fatty Acid Synthase
HAPs: Hypoxia-activated Prodrugs
